# Knowledge and awareness of artificial intelligence among dental OPD patients at Patna medical college hospital

**DOI:** 10.6026/973206300220817

**Published:** 2026-02-28

**Authors:** Imran Md, Vaibhava Raaj, Subhash Kumar, Qalbi Fatima, Pallavi Priya, Abhishek Sinha

**Affiliations:** 1 Department of Dentistry, Patna Medical College & Hospital, Patna, Bihar, India

**Keywords:** Artificial intelligence, patient awareness, dentistry, digital health, patient education, healthcare technology

## Abstract

Limited patient understanding of artificial intelligence may hinder its effective integration into dental healthcare delivery. Therefore, it is of interest to assess knowledge, awareness and attitudes toward artificial intelligence among 420 patients attending the dental outpatient department of Patna Medical College and Hospital, September 2025 to Janurary 2026 using a validated structured questionnaire. Although 72.4% of participants had heard of artificial intelligence, only 28.6% demonstrated adequate knowledge of its healthcare applications and just 18.3% were aware of specific dental AI technologies. Awareness scores were significantly influenced by age, education level and urban residence (p < 0.001), while major concerns included data privacy, replacement of dentists and diagnostic reliability. Thus, we show substantial gaps in patient knowledge, underscoring the need for targeted educational initiatives to support informed acceptance of artificial intelligence in dental practice.

## Background:

Artificial intelligence is one of the most innovative changes in technology in the modern healthcare sector and it has been used in diagnostic image interpretation, treatment planning, prognostic prediction and clinical decision support systems [1]. The adoption of AI technologies in medical and dental practice has increased significantly in the last 10 years due to the development of machine learning algorithms and the growth of computing power and the availability of digital health data [2]. In the field of dentistry specifically, artificial intelligence applications have been found to perform exceptionally well in various areas of detection of caries on radiographic images, periodontal diseases, oral cancer screening, orthodontic treatment planning as well as optimal placement of implants [3]. The diagnostic accuracy of deep learning algorithms has reached or even surpassed that of trained clinicians in some image-based tasks and it is proposed that there is a major potential to improve diagnostic accuracy and minimize practitioner variability [4]. The technical performance of AI technologies in clinical settings is not the only factor that influences their successful implementation, as it needs to be accepted by both the medical professionals and the patients [5]. Edification of patient attitudes to AI- assisted healthcare delivery determines the treatment adherence, satisfaction and general engagement with healthcare services. The views of patients towards these new technologies are also critical, as this understanding would enable relevant implementation strategies [6]. Surveys in the world have shown that there is a wide range of differences in the awareness and acceptance of healthcare AI due to the culture, level of education, previous experience with technology and the nature of healthcare systems [7]. Research studies in developed countries have recorded moderate to high awareness but still citing poor concerns on aspects of privacy, accuracy as well as the possibility of reducing the human interaction in healthcare encounter [8]. The Indian healthcare environment also has its own set of considerations when it comes to implementing AI adoption that is marked by high disparities in access to technology by urban and rural population, the variety of educational background of patients and digital illiteracy [9]. Whereas exposures to digital health technologies have been on the rise in metropolitan centers, in smaller cities and rural areas, awareness of the same might be significantly lower [10]. Being among the most populated states of India, Bihar has a large number of patients that the public healthcare infrastructure serves the populations. Patna Medical College and Hospital is one of the leading tertiary care hospitals that offer dental treatment to various categories of patients including urban, semi-urban and rural demographics [11]. The knowledge of patient awareness, as well as their attitudes to AI, in that regard is a good input to consider when planning the implementation of technology. The literature addressing patient attitudes toward dental AI is very sparse, with the available sources about the issue mostly concentrating on the attitudes of clinicians and technical validation of medical AI applications [12]. There has been minimal consideration of the patient perspective especially in the developing countries contexts even though it is of paramount significance in the ultimate adoption of technology [13]. A number of factors might impact patient awareness and acceptance of AI in healthcare, such as age, education, occupation, previous experience with digital technologies and exposure to health information via different media channels among others [14]. Recognition of such determinants allows designing education approaches that focus on the particular areas of knowledge deficit and issues [15]. Therefore, it is of interest to evaluate the knowledge, awareness and attitudes toward artificial intelligence among patients attending the dental outpatient department of a tertiary care hospital in Bihar.

## Materials and Methods:

## Study design and setting:

This descriptive cross-sectional study was conducted at the Outpatient Department of Dentistry, Patna Medical College & Hospital, Patna, Bihar, India, between September 2025 to Janurary 2026. Patna Medical College & Hospital is a premier tertiary care teaching institution providing comprehensive dental services to patients from Patna city and surrounding districts of Bihar.

## Ethical considerations:

The study protocol was approved by the Institutional Ethics Committee of Patna Medical College & Hospital prior to commencement. Written informed consent was obtained from all participants following detailed explanation of study objectives, procedures and confidentiality assurances. Participation was entirely voluntary, with no impact on dental treatment provided.

## Sample size determination:

Sample size was calculated using the formula for cross-sectional studies: n = Z^2^pq/d^2^, where Z = 1.96 (95% confidence level), p = 0.50 (anticipated proportion with AI awareness, conservatively estimated), q = 1-p and d = 0.05 (margin of error). This yielded a minimum requirement of 384 participants. Accounting for incomplete responses and non-participation, a target sample of 450 was established.

## Participant selection:

Consecutive sampling was employed, with eligible patients visiting the dental OPD during the study period invited to participate.

## Inclusion criteria:

[1] Age 18 years or above

[2] Ability to comprehend Hindi or English

[3] Willingness to provide informed consent

[4] Sufficient time available to complete questionnaire

[5] No acute dental emergency requiring immediate treatment

## Exclusion criteria:

[1] Cognitive impairment affecting questionnaire comprehension

[2] Previous participation in the study

[3] Inability to provide informed consent

[4] Healthcare professionals or medical/dental students

## Study instrument:

A structured questionnaire was developed based on review of existing validated instruments for assessing health technology awareness, adapted for the dental AI context. The questionnaire underwent content validation by a panel of five experts comprising dental professionals, public health specialists and health communication researchers. Pilot testing was conducted among 30 patients (excluded from final analysis) to assess comprehensibility, with minor modifications incorporated based on feedback.

## The final questionnaire comprised 35 items organized into six sections:

## Section A:

## Demographic Information (8 items):

Age, gender, educational qualification, occupation, residence type, monthly household income, smartphone usage and internet access.

## Section B:

## General AI Awareness (6 items):

Prior exposure to AI concept, information sources, self-perceived familiarity and understanding of basic AI principles.

## Section C:

## Healthcare AI Knowledge (7 items):

Awareness of AI applications in medicine and dentistry, specific examples, perceived current usage and accuracy perceptions.

## Section D:

## Attitudes and Acceptance (6 items):

Willingness to receive AI-assisted diagnosis, comfort levels, trust perceptions and preference between AI and human practitioners.

## Section E:

## Concerns and Barriers (5 items):

Privacy concerns, accuracy doubts, fear of technology replacement, cost considerations and accessibility issues.

## Section F:

## Information needs (3 items):

Desire for additional information, preferred education formats and willingness to learn about dental AI. Response formats included dichotomous (yes/no), multiple choice and 5-point Likert scale items. A knowledge score was computed based on correct responses to factual questions, with scores categorized as: poor (0-4), moderate (5-8) and good (9-13).

## Data collection procedure:

Trained research assistants approached eligible patients in the waiting area of the dental OPD. Following consent, participants completed the self-administered questionnaire in a designated area ensuring privacy. For participants with limited literacy, research assistants administered the questionnaire orally in Hindi without leading or influencing responses. Average completion time was 12-15 minutes. Completed questionnaires were reviewed for completeness before acceptance.

## Statistical analysis:

Data were entered into Microsoft Excel and analysed using SPSS version 25.0. Continuous variables were expressed as mean ± standard deviation, while categorical variables were presented as frequencies and percentages. Chi-square test assessed associations between categorical variables. Independent samples t-test and one-way ANOVA compared mean scores across groups. Pearson correlation examined relationships between continuous variables. Multiple linear regressions identified independent predictors of AI knowledge scores. Statistical significance was defined as p < 0.05.

## Results:

Of 450 patients approached, 420 completed the questionnaire satisfactorily, yielding a response rate of 93.3%. The study population comprised 234 males (55.7%) and 186 females (44.3%), with mean age of 38.6 ± 13.8 years (range: 18-72 years). Educational distribution showed considerable variation, with 12.4% having completed higher education, 34.3% secondary education, 31.9% primary education and 21.4% with no formal education or minimal literacy. Regarding residence, 48.6% were urban dwellers, 28.3% semi-urban and 23.1% rural. Smartphone ownership was reported by 68.8%, with 54.3% having regular internet access. The majority (78.6%) were visiting for general dental complaints, while 21.4% presented for specific procedures or follow-up (Table 1). General awareness of artificial intelligence was reported by 304 participants (72.4%), having heard the term through various sources. Television and films constituted the primary information source (42.8%), followed by social media (28.6%), newspapers/magazines (12.5%), family/friends (10.2%) and formal education (5.9%). However, awareness of AI applications in healthcare was considerably lower, with only 168 participants (40.0%) aware that AI could be used in medical settings. Specific awareness of dental AI applications was reported by merely 77 participants (18.3%), with caries detection being the most recognized application (8.6%), followed by orthodontic planning (4.8%) and oral cancer screening (3.1%). Mean knowledge score was 4.8 ± 3.2 out of maximum 13 points. Knowledge was categorized as poor in 248 participants (59.0%), moderate in 132 (31.4%) and good in 40 (9.5%) (Table 2). Among participants with AI awareness (n=304), attitudes toward AI-assisted dental care were mixed. While 156 (51.3%) expressed willingness to receive AI-assisted diagnosis if recommended by their dentist, only 82 (27.0%) indicated comfort with AI making treatment decisions without human oversight. Trust in AI accuracy was reported by 124 (40.8%), with 98 (32.2%) neutral and 82 (27.0%) expressing distrust. Multiple concerns regarding dental AI were identified. Data privacy emerged as the predominant concern (68.2%), followed by fear of AI replacing human dentists (54.8%), doubts about diagnostic accuracy (47.6%), increased treatment costs (42.4%) and reduced personal interaction with healthcare providers (38.6%). When presented with choice scenarios, 268 participants (63.8%) preferred human dentists exclusively, 112 (26.7%) preferred AI-assisted human care and 40 (9.5%) expressed no preference. None expressed preference for AI-only care (Table 3). Multiple linear regression analysis identified independent predictors of AI knowledge scores. Educational attainment emerged as the strongest predictor (β = 0.426, p < 0.001), followed by internet access (β = 0.218, p < 0.001), younger age (β = -0.186, p < 0.001) and urban residence (β = 0.142, p = 0.002). Gender and income level did not remain significant after adjustment. The model explained 52.4% of variance in knowledge scores (adjusted R^2^ = 0.524, F = 68.42, p < 0.001). Correlation analysis revealed significant positive associations between knowledge scores and willingness to accept AI-assisted care (r = 0.384, p < 0.001) and trust in AI accuracy (r = 0.326, p < 0.001), while negative correlation existed with overall concern intensity (r = -0.298, p < 0.001).

## Discussion:

The paper gives a good understanding of patient awareness, perception of artificial intelligence in dental care in a large public tertiary care hospital in Bihar, India. These results show that there are quite enormous knowledge gaps and that there are a lot of concerns that have to be resolved to enable successful implementation of AI technologies in the dental practice. The overall level of awareness of the term artificial intelligence is 72.4%, which is quite promising, but the degree of awareness of the applications in the healthcare context is significantly lower. The same trends have already been recorded in other settings on developing countries where exposure through media induces a shallow familiarity as opposed to an understanding of the media content [16]. The fact that 18.3% of the respondents are aware of dental AI applications points specifically to the fact that the communication about dental AI technologies has not been as effective as it needs to be when it comes to reaching patient populations in particular [17]. TV and movies became the most common source of information about AI, as is expected under other circumstances, where entertainment media influences the attitude of the population towards new technologies [18]. Nevertheless, fictional representations can cause misunderstandings of AI capabilities and risks, which can become the basis of unrealistic expectations or unjustified fears [19]. The small contribution of formal education channels to transmission of AI awareness points to missed chances of proper health technology literacy promotion. The close correlation between AI knowledge and education level in the present research is consistent with the reported results in various global settings [20]. Education not only has direct effect on the acquisition of knowledge but also on behavior of seeking information and critical assessment of information concerning technology [21]. The low awareness among the individuals who are not educated is especially low and, as a result, the necessity of incorporating education strategies that do not involve any literacy issues. The difference in AI awareness based on age represents differences in how a technology was exposed and adopted across different global generations [22]. The familiarity of younger adults with digital technologies is also more occupationally, educationally and socially oriented, which makes it easier to be receptive to new applications such as healthcare AI [23]. Nonetheless, since older patients tend to have more healthcare demands, special outreach must be provided to them to enable equal access to AI-enhanced services. The urban to rural awareness gap recorded in this research is indicative of the bigger trends in digital divide in India, where the infrastructure access and lower penetration of technologies in rural locations limit the exposure of rural population to digital health innovations [24]. Since large shares of the patients of the state institutions, such as Patna Medical College & Hospital, live in the rural regions, the filling of this awareness gap means a specific significance [25]. The most common hindrance to AI adoption was the issue of privacy, which was raised by more than two-thirds of respondents. This observation aligns with the global literature that has reported that people have always been deeply concerned with the safety of their health data under the influence of AI systems that need to handle vast amounts of data [26]. The recent data breaches in other industries on high profiles can increase these fears even among those groups with low direct AI exposure [27]. This fear of AI practitioners becoming the next human healthcare provider among more than half of the respondents is indicative of the entrenched cultural and psychological aspects of wanting to deal with human beings in the sphere of healthcare [28]. Communication should also focus on the supportive and not the substitutive role of AI technologies, where they are seen as an aid instead of a substitute to the dentist-patient relationship [29]. Essentially, accuracy issues, though not as widespread as privacy concerns, should be addressed as they may affect treatment acceptance. Research on other healthcare sectors has recorded the fact that willingness to adhere to AI-generated recommendations is highly reliant on perceptions of reliability [30]. The confidence should be developed by means of clear communication on AI performance attributes and proper use cases [31]. The overwhelming focus on human practitioners with none of the participants supporting AI-only patient care establishes the fact that human relationships remain central in the delivery of healthcare despite technological progress [32]. This observation confirms the implementation models that focus on AI as an addition to professional practice, but not as autonomous system, which is in line with the existing regulatory and ethical frameworks [33]. The existence of a positive correlation between knowledge and acceptance indicates that barriers to the adoption of AI can be successfully tackled by using educational interventions. There are also reported similar relations in other contexts of health technology, which justify the investment in patient education programs [34]. The fact that three-quarters of the participants expressed the desire to get more information implies the willingness to get such educational activities. The preferred position of the participants to the use of verbal explanation by the dentists as the main information format demonstrates the credibility of the healthcare providers in their communication with technology [35]. The education of dental professionals in order to train them to communicate with patients on the use of AI applications should therefore be a priority along with patient-led education programs. Limitations of the study are that the single-institution location might have restricted the generalizability of the study results, the design was cross-sectional and could not evaluate any change in awareness with time and there was a possibility that the attitudes were reported with social desirability. Also, the dynamic state of AI technology implies that the patterns of awareness can change quickly due to the changing media reports and the implementation of the technologies. The results can be applied to the AI implementation planning in the dental healthcare facilities that work with the similar populations. Patient education prior to implementation, focused on certain issues as revealed by this study, could enable acceptance and use [36]. There should be a focus on the development of culturally suitable educational resources in local languages by use of available formats [37].

## Conclusion:

We show that although general awareness of artificial intelligence exists among dental patients at Patna Medical College and Hospital, detailed knowledge of its healthcare and dental applications remains limited. Educational level, age, internet use and place of residence significantly influenced awareness, while concerns regarding data privacy, replacement of dentists and diagnostic accuracy were major barriers to acceptance. Thus, we show the need for targeted patient education and effective dentist-patient communication to support informed acceptance of artificial intelligence in dental care.

## Figures and Tables

**Table 1:** Sociodemographic characteristics of study participants (n=420)

**Characteristic**	**Frequency**	**Percentage (%)**
**Age Group**		
18-30 years	142	33.8
31-45 years	148	35.2
46-60 years	98	23.3
>60 years	32	7.6
**Gender**		
Male	234	55.7
Female	186	44.3
**Educational Qualification**		
No formal education	90	21.4
Primary education	134	31.9
Secondary education	144	34.3
Higher education	52	12.4
**Occupation**		
Unemployed/Homemaker	118	28.1
Farmer/Agricultural worker	68	16.2
Business/Self-employed	86	20.5
Private sector employee	78	18.6
Government employee	42	10
Student	28	6.7
**Residence Type**		
Urban	204	48.6
Semi-urban	119	28.3
Rural	97	23.1
**Monthly Household Income**		
<₹15,000	156	37.1
₹15,000-30,000	138	32.9
₹30,000-50,000	82	19.5
>₹50,000	44	10.5
**Smartphone Ownership**		
Yes	289	68.8
No	131	31.2
**Regular Internet Access**		
Yes	228	54.3
No	192	45.7

**Table 2:** AI knowledge and awareness levels stratified by demographics

**Variable**	**n**	**Mean Knowledge Score±SD**	**Adequate Awareness n (%)**	**p-value**
Overall	420	4.8±3.2	120 (28.6)	-
Age Group				<0.001
18-30 years	142	6.4±3.1	58 (40.8)	
31-45 years	148	5.2±3.0	42 (28.4)	
46-60 years	98	3.4±2.6	16 (16.3)	
>60 years	32	2.1±1.8	4 (12.5)	
Gender				0.024
Male	234	5.2±3.3	76 (32.5)	
Female	186	4.3±3.0	44 (23.7)	
Education				<0.001
No formal education	90	1.8±1.4	4 (4.4)	
Primary education	134	3.6±2.2	18 (13.4)	
Secondary education	144	5.8±2.8	52 (36.1)	
Higher education	52	8.6±2.4	46 (88.5)	
Residence				<0.001
Urban	204	6.2±3.2	82 (40.2)	
Semi-urban	119	4.2±2.6	28 (23.5)	
Rural	97	2.8±2.1	10 (10.3)	
Income Level				<0.001
<₹15,000	156	3.2±2.4	22 (14.1)	
₹15,000-30,000	138	4.8±2.8	36 (26.1)	
₹30,000-50,000	82	6.4±3.1	38 (46.3)	
>₹50,000	44	7.8±3.0	24 (54.5)	
Smartphone Ownership				<0.001
Yes	289	5.6±3.2	102 (35.3)	
No	131	3.1±2.4	18 (13.7)	
Internet Access				<0.001
Yes	228	6.2±3.1	96 (42.1)	
No	192	3.1±2.4	24 (12.5)	

**Table 3:** Attitudes, concerns and acceptance regarding dental AI (n=420)

**Parameter**	**n**	**Percentage (%)**
**Willingness to Accept AI-Assisted Diagnosis**		
Yes, definitely	86	20.5
Yes, if dentist recommends	156	37.1
Unsure	94	22.4
No	84	20
**Trust in AI Diagnostic Accuracy**		
High trust	48	11.4
Moderate trust	124	29.5
Neutral	98	23.3
Low trust	96	22.9
No trust	54	12.9
**Comfort with AI Treatment Decisions**		
Very comfortable	24	5.7
Somewhat comfortable	82	19.5
Neutral	108	25.7
Somewhat uncomfortable	124	29.5
Very uncomfortable	82	19.5
**Primary Concerns (Multiple Responses)**		
Data privacy and security	286	68.2
AI replacing human dentists	230	54.8
Accuracy and error risk	200	47.6
Increased treatment costs	178	42.4
Reduced human interaction	162	38.6
Technology malfunction risk	148	35.2
Lack of accountability	126	30
**Practitioner Preference**		
Human dentist only	268	63.8
AI-assisted human care	112	26.7
No preference	40	9.5
AI only	0	0
**Desire for More AI Information**		
Yes	312	74.3
No	68	16.2
Unsure	40	9.5
**Preferred Information Format**		
Verbal explanation by dentist	186	44.3
Video demonstration	112	26.7
Printed brochures	64	15.2
Online resources	42	10
Group education sessions	16	3.8
